# Long-term effects of a preconception lifestyle intervention on cardiometabolic health of overweight and obese women

**DOI:** 10.1093/eurpub/cky222

**Published:** 2018-10-30

**Authors:** Vincent Wekker, Emilia Huvinen, Lotte van Dammen, Kristiina Rono, Rebecca C Painter, Aeilko H Zwinderman, Cornelieke van de Beek, Taisto Sarkola, Ben Willem J Mol, Henk Groen, Annemieke Hoek, Saila B Koivusalo, Tessa J Roseboom, Johan G Eriksson

**Affiliations:** 1 Department of Obstetrics and Gynaecology, Amsterdam UMC, University of Amsterdam, Amsterdam, The Netherlands; 2 Amsterdam Reproduction and Development Research Institute, Amsterdam UMC, Amsterdam, The Netherlands; 3 Department of Clinical Epidemiology, Biostatistics and Bioinformatics, Amsterdam UMC, University of Amsterdam, Amsterdam, The Netherlands; 4 Amsterdam Public Health Research Institute, Amsterdam UMC, Amsterdam, The Netherlands; 5 Department of Obstetrics and Gynaecology, University of Helsinki, Helsinki University Hospital, Helsinki, Finland; 6 Department of Obstetrics and Gynaecology, University of Groningen, University Medical Centre Groningen, Groningen, The Netherlands; 7 Department of Epidemiology, University of Groningen, University Medical Centre Groningen, Groningen, The Netherlands; 8 Children’s Hospital, Helsinki University Hospital, Helsinki, Finland; 9 Department of Obstetrics and Gynaecology, Monash Medical Centre, Monash Health and Monash University, Clayton, Australia; 10 Unit of General Practice and Primary Health Care, University of Helsinki, Helsinki University Hospital, Helsinki, Finland; 11 Folkhälsan Research Center, Helsinki, FInland

## Abstract

**Background:**

The global prevalence of obesity in women keeps increasing. The preconception period may be a window of opportunity to improve lifestyle, reduce obesity and improve cardiometabolic health. This study assessed the effect of a preconception lifestyle intervention on long-term cardiometabolic health in two randomized controlled trials (RCTs).

**Methods:**

Participants of the LIFEstyle and RADIEL preconception lifestyle intervention studies with a baseline body mass index (BMI) ≥29 kg/m^2^ were eligible for this follow-up study. Both studies randomized between a lifestyle intervention targeting physical activity, diet and behaviour modification or usual care. We assessed cardiometabolic health 6 years after randomization.

**Results:**

In the LIFEstyle study (*n* = 111) and RADIEL study (*n* = 39), no statistically significant differences between the intervention and control groups were found for body composition, blood pressure, arterial stiffness, fasting glucose, homeostasis model assessment of insulin resistance, HbA1c, lipids and high sensitive C-reactive protein levels 6 years after randomization. Participants of the LIFEstyle study who successfully lost ≥5% bodyweight or reached a BMI <29 kg/m^2^ during the intervention (*n* = 22, [44%]) had lower weight (−8.1 kg; 99% CI [−16.6 to −0.9]), BMI (−3.3 kg/m^2^; [−6.5 to −0.8]), waist circumference (−8.2 cm; [−15.3 to −1.3]), fasting glucose (−0.5 mmol/L; [−1.1 to −0.0]), HbA1c (−4.1 mmol/mol; [−9.1 to −0.8]), and higher HDL-C (0.3 mmol/L; [0.1–0.5]) compared with controls.

**Conclusion:**

We found no evidence of improved cardiometabolic health 6 years after a preconception lifestyle intervention among overweight and obese women in two RCTs. Women who successfully lost weight during the intervention had better cardiometabolic health 6 years later, emphasizing the potential of successful preconception lifestyle improvement.

## Introduction

The prevalence of obesity continues to increase globally.^1^ Obesity is a major modifiable risk factor for cardiometabolic disease.^2^ Among women, obesity increases the risk of pregnancy complications which also increases the risk of cardiovascular disease.^3^

Lifestyle interventions targeting diet, physical activity and behaviour are recommended as treatment for obesity.^4^ These interventions have the potential to decrease weight, lower the risks of cardiometabolic diseases, but also reduce the risks of pregnancy-associated complications.^5^^,^^6^ Because women who are planning a pregnancy are more susceptible to lifestyle advice, the preconception period might be an optimal window of opportunity for a lifestyle intervention.^7–9^ Preconception lifestyle interventions can improve lifestyle, induce weight loss and improve spontaneous pregnancy rates and outcomes.^10–12^ We previously showed that a 6-month preconception lifestyle intervention in obese infertile women improved cardiometabolic health, halved the odds for metabolic syndrome (MetS), and increased quality of life, during and directly after the intervention period.^13^

Although these short-term effects are promising, previous studies have shown that permanent lifestyle changes are difficult to achieve and many people regain weight over time.^14^ Therefore, we aimed to assess the long-term effects of a preconception lifestyle intervention on cardiometabolic health, based on the follow-up of the Dutch ‘LIFEstyle’ and Finnish ‘RADIEL’ preconception lifestyle intervention trials.^15^^,^^16^

## Methods

### The randomized controlled trials

The protocols of the LIFEstyle (NTR 1530) and RADIEL (IDr: NCT01698385) studies have been published previously and had ethical approval.^15^^,^^16^ Both studies were preconception lifestyle interventions among overweight or obese women (table 1). All participants provided written informed consent.

**Table 1:** Comparison of lifestyle interventions applied in the LIFEstyle and RADIEL study

	LIFEstyle study	RADIEL study
**Inclusion criteria**	Age 18–39 years BMI ≥29 kg/m^2^ Chronic anovulation or infertility >1 year	Age >18 years BMI ≥30 kg/m^2^ and/or prior GDM
**Goal**	5–10% weight reduction BMI <29 kg/m^2^	5% pre-pregnancy weight reduction No gestational weight gain during first and second trimester of pregnancy
**Amount of consultations**	Max. 10 consultations in the 6 months before fertility treatment: 6 outpatient clinic visits 4 telephone consultations	Max. 14 consultations: Every 3 months before pregnancy (max. 5 visits) 3 times in pregnancy (1/trimester) 3 times after pregnancy (6 weeks, 6 months and 12 months postpartum) 3 group sessions (baseline, 6 and 12 months postpartum)
**Diet**	Reduction of caloric intake of 600 kcal/day, with a minimum intake of 1200 kcal/day using an online diary. Improve diet quality.	Reduction of caloric intake to 1600–1800 kcal/day. Using the plate model (40–50% carbohydrates, 30–40% fats and 20–25% proteins). Improve diet quality.
**Physical activity**	Daily physical activity aimed at 10 000 steps per day using a pedometer. Moderate-intensity physical activity for at least 30 min, 2–3 times a week.	Daily physical activity aimed at 10 000 steps per day, using a pedometer. Moderate intensity physical activity for 150 min/week.
**Motivational counselling**	Individualized motivational counselling: ^ Awareness of actual lifestyle leading to overweight or obesity. ^ Awareness of healthy lifestyle in relation to infertility. ^ Formulating individualized goals embedded in a ‘patient contract’. Motivation to change physical activity was monitored by the ‘Physician-based Assessment and Counselling for Exercise’ and counselling was adjusted accordingly.	Individualized motivational counselling based on personal preferences. Contact with local physical activity counsellor and entry tickets for local sport clubs. Goals were adapted to pregnancy on individual basis.

#### The initial LIFEstyle study

The LIFEstyle study, a multi-centre randomized controlled trial (RCT), was conducted in 23 medical centres in the Netherlands between 2009 and 2014. Infertile women between 18 and 39 years of age with a body mass index (BMI) ≥29 kg/m^2^ were eligible for inclusion. Infertility was defined as chronic anovulation or unsuccessful conception for over 12 months.

Women were successfully randomized (1: 1) to a 6-month lifestyle intervention preceding infertility treatment (intervention group) or prompt infertility treatment according the Dutch guidelines (control group) irrespective of BMI, stratified for trial centre and ovulatory status.^17^^,^^18^

The LIFEstyle intervention led by trained intervention coaches consisted of six 30-min face-to-face sessions at the outpatient clinics and four by telephone or e-mail, aiming at 5–10% weight reduction or a BMI <29 kg/m^2^ within 6 months. Women reaching this goal did not have to finish the 6-month intervention, but could proceed with conventional infertility treatment. The intervention consisted of a dietary, physical activity and behavioural modification component.^19^ The dietary component was supported with an online diary and aimed at caloric reduction of 600 kcal with a minimum intake of 1200 kcal/day. The physical activity component aimed at moderate-intensity physical activity for at least two or three times a week and 10 000 steps/day, stimulated with the use of a pedometer. The behavioural modification part of the intervention was focussed on creating awareness of lifestyle predisposing to obesity, and the goals were determined on individual basis. The intervention stopped in case of pregnancy, but was resumed after a miscarriage within 6 months after randomization.

Women in the control group were given written information about the negative effects of overweight/obesity on fertility, as part of the usual care.

#### The initial RADIEL study

The RADIEL study was a multi-centre RCT, conducted in four maternity hospitals in Finland, between 2008 and 2013. Women ≥18 years of age who were planning to become pregnant, with a BMI ≥30 kg/m^2^ and/or a history of gestational diabetes (GDM) were eligible for inclusion. Women who met these criteria were recruited through advertisements in newspapers, social media and antenatal clinics, as well as by personal invitation letters based on the hospital record information on their history of GDM.

Women were successfully randomized to a lifestyle intervention (intervention group) or usual care (control group), stratified for trial centre and risk factors (history of GDM or preconception BMI ≥30 kg/m^2^).^20^ The intervention group received a structured lifestyle intervention consisting of a maximum of 11 face-to-face sessions and 3 group sessions, provided by trained study nurses. The individual sessions were scheduled every 3 months before pregnancy, once in each trimester of pregnancy, and 6 weeks, 6 and 12 months postpartum. The aim of the intervention was 5% weight reduction and no gestational weight gain in the first and second trimesters. Dietary advice was based on Nordic dietary recommendations encouraging use of vegetables, berries and fish and avoiding sugar-rich foods and saturated fat.^21^^,^^22^ The recommendation for caloric intake was 1600–1800 kcal/day with 40–50% of total energy (E%) coming from carbohydrates, 30–40 E% from fats and 20–25 E% from proteins. Participants also attended group sessions led by a dietitian at the enrolment, during the first trimester, and 6 and 12 months after delivery. Physical activity goal was 150 min of moderately strenuous exercise per week. The participants received pedometers and were encouraged to reach 10 000 steps/day. Lifestyle advice was personalized according to individual preferences and pregnancy status. Women in the control group received general information leaflets about diet and physical activity.

### The follow-up studies

#### The LIFEstyle follow-up study

Women who participated in the original LIFEstyle study and who were not lost to follow-up were eligible for the follow-up study. The follow-up was performed from April 2016 until August 2017. A physical assessment after a 2-h fast was performed minimally 6 months after pregnancy, under standardized conditions, inside of a mobile research vehicle close to the participants’ homes. Blood samples were taken at home, during a separate visit by a research nurse, after an overnight fast. Biochemical analyses were performed by the AMC Clinical Chemistry Laboratory for the biochemical analyses.^23^

#### The RADIEL follow-up study

Women who gave birth after participation in the original study and who had at least one study visit during pregnancy were approached for the follow-up study. The follow-up was performed from May 2014 until April 2017. The physical assessment took place at the Folkhälsan Research Center in Helsinki and Lappeenranta at the South Karelian Central Hospital. Anthropometric measurements and blood samples were taken during the study visit after a 10–12 h overnight fast. Biochemical analyses were performed by the HUSLAB central laboratory in Helsinki and Central Hospital laboratory in Lappeenranta. The current study includes women who entered the RADIEL study prior to pregnancy with a BMI ≥29 kg/m^2^.

### Outcomes

Assessments included weight (LIFEstyle study: SECA 877; RADIEL: InBody720), height (SECA 206), and waist and hip circumferences (LIFEstyle: SECA 201; RADIEL: Prym). BMI was calculated as (weight [kg]/length [m^2^]). Systolic and diastolic blood pressure was measured in sitting position using oscillometry (LIFEstyle: OMRON HBP-1300; RADIEL: OMRON M6W Intellisense). We assessed fasting concentrations of triglycerides, total cholesterol, low-density lipoprotein cholesterol (LDL-C), high-density lipoprotein cholesterol (HDL-C) and high sensitive C-reactive protein (hs-CRP), fasting glucose and insulin. The homeostasis model assessment of insulin resistance (HOMA-IR) was calculated ([fasting insulin (mU/l)] × [fasting glucose (mmol/l)]/22.5).^24^

Pulse wave velocity (PWV), a marker for aortic elasticity, was measured between the carotid and femoral artery with mechanotransducer sensors using the Complior (ALAM Medical, France) at rest and in supine position. To calculate PWV the following equation was used: PWV = 0.8 × (direct distance between a. carotis and a. fermoralis measuring site/Δ time between upstroke of pressure waves). A scaling factor of 0.8 was used because direct distance leads to overestimation of real PWV.^25^ Body fat percentage (BFP) was measured in the RADIEL study with multi-frequency bio-impedance measurement method using the InBody 3.0 (Biospace Co, Ltd, Seoul, Korea), and in the LIFEstyle study BFP was measured with arm-to-leg bioelectrical impedance analysis using the Bodystat 1500 (Bodystat Ltd, Isle of Man, UK) and the fat-free mass prediction equation by Kyle *et al.*^26^ All outcomes were measured two times. In the LIFEstyle study outcomes were measured a third time in case of substantial differences (>1 cm or >10%) between the first two measurements. The mean of the measurements is used in the statistical analyses.

Participants were identified with MetS based on the 2001 revised criteria of the National Cholesterol Education Programme ATP III.^27^ Participants had to meet at least three of the following criteria: (i) plasma glucose ≥5.6 mmol/L or known drug treatment for elevated blood glucose; (ii) HDL-C < 1.3 mmol/L or known drug treatment for low HDL cholesterol; (iii) triglycerides ≥1.7 mmol/L or known drug treatment for elevated triglycerides; (iv) waist circumference ≥88 cm or (v) blood pressure ≥130/85 mmHg or known drug treatment for elevated blood pressure.

### Statistical analyses

Participants were analysed on intention-to-treat basis, in the treatment group in which they were originally randomized. For comparison of the baseline variables independent student t-tests or Mann–Whitney-U tests were performed for continuous variables and Chi-Square tests for binary or categorical variables. The continuous outcomes were analysed with linear regression, including 1500 bootstrap samples to calculate 99% bias-corrected and accelerated confidence intervals (99% BCa CI), because the normality assumption of linear regression appeared to be violated for some of the outcome variables (IBM SPSS version 24.0, Armonk, NY, USA). The regression models included the outcomes of interest as the dependent and the treatment group as independent factor. If available, baseline values of the outcomes of interests and potential confounders that differed between the treatment groups were incorporated as covariates. The difference between the groups was considered statistically significant if the CI of the mean difference did not include zero. Mixed effect logistic regression analyses including baseline and follow-up data were performed for binary outcomes (STATA version 15.0, College Station, TX, USA). The intervention effect was assessed by the interaction between time and treatment group. Subgroup analyses were performed to compare successful women with the control group. Women were identified as ‘successful’ if they had reduced their body weight ≥5% or lowered their BMI under 29 kg/m^2^. Baseline differences in characteristics between the successful women and the control group were added as covariates to the adjusted model. Further explorative analyses were performed to assess (i) the interaction between treatment group and pregnancy status after randomization as well as (ii) between treatment group and polycystic ovary syndrome (PCOS) status on the outcomes of interest.^28^ These explorative analyses were only performed in the LIFEstyle study population, because of data availability.

## Results

### Participation

**Figure 1:**
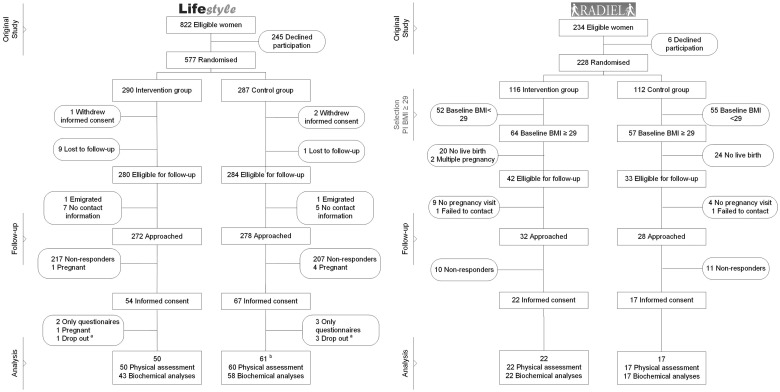
Flow-chart of study participants. a: One woman in the intervention and three women in the control group had given informed consent but cancelled the physical measurement visit. b: One woman only attended the blood sample collection, but cancelled the physical measurement.

The flow charts of both studies are presented in Figure 1. Of the 577 women who participated in the LIFEstyle study, 111 (19.3%) with a median follow-up duration of 73.1 months (interquartile range [IQR] 63.9–80.4) were included in the current study, of whom 50 women were included in the intervention and 61 in the control group. Of the 234 women who were recruited before pregnancy in the RADIEL study, 121 women had a BMI of 29.0 or above. Of these eligible women 39 (32.2%) with a median follow-up duration of 74.2 months (IQR 70.7–81.8) were included in this study, of whom 22 women were included in the intervention and 17 in the control group.

### Characteristics of participants

Baseline and follow-up characteristics of the participating women in both studies are presented in table 2. A total of 82 (74%) women of the LIFEstyle study, in comparison to all women of the RADIEL study had a pregnancy of more than 24 weeks between randomization and follow-up.

**Table 2:** Baseline and follow-up characteristics of the study participants

*Baseline characteristics*	LIFEstyle Study			RADIEL study		
Variables	Intervention (*n* = 50)	Control (*n* = 61)	*P*-value^a^	Intervention (*n* = 22)	Control (*n* = 17)	*P*-value^a^
Age, years—mean (SD)	30.4 (4.0)	30.4 (4.2)	0.94	34.2 (3.3)	31.6 (5.3)	0.09
Ethnicity—no. Caucasian (%)	48 (96)	57 (93.4)	0.69	22 (100)	17 (100)	n.a.
Education—no. (%)			0.95			0.41
Basic education	0 (0)	1 (1.7)		0 (0)	0 (0)	
Vocational education	11 (23.4)	12 (20.3)		4 (19.0)	2 (11.8)	
Secondary education	1 (2.1)	1 (1.7)		1 (4.8)	4 (23.5)	
Vocational & Secondary education	24 (51.1)	28 (47.5)		9 (42.9)	7 (41.2)	
Higher education	11 (23.4)	17 (28.8)		7 (33.3)	4 (23.5)	
Alcohol use—no. (%)	19 (44.2)	19 (32.8)	0.24	11 (52.4)	12 (70.6)	0.25
Current smoker—no. (%)	14 (28.6)	11 (18.0)	0.19	1 (4.5)	1 (5.9)	1.00
Nulliparous—no. (%)	41 (82.0)	45 (73.8)	0.30	2 (9.1)	5 (29.4)	0.21
Weight, kg—mean (SD)	103.6 (13.0)	102.8 (11.4)	0.75	97.5 (15.0)	94.7 (16.4)	0.59
BMI, kg/m^2^—mean (SD)	35.5 (2.9)	35.8 (3.2)	0.64	35.2 (4.0)	34.0 (5.2)	0.44
Systolic blood pressure, mmHg—mean (SD)	123.1 (11.8)	127.2 (12.5)	0.08	127.3 (9.6)	120.3 (9.6)	0.03
Diastolic blood pressure, mmHg—mean (SD)	79.0 (8.3)	81.6 (7.2)	0.08	85.1 (8.5)	80.8 (8.0)	0.12
Fasting glucose, mmol/L—mean (SD)	5.1 (0.3)	5.5 (0.9)	0.02	5.6 (0.5)	5.6 (0.4)	0.82
HOMA-IR—median (IQR)	2.9 (1.8–3.9)	3.1 (2.4–4.2)	0.26	2.3 (1.8–3.5)	2.3 (1.5–4.4)	0.78
Total Cholesterol, mmol/L—mean (SD)	4.8 (1.0)	4.7 (0.8)	0.56	4.8 (0.6)	4.7 (1.0)	0.57
LDL Cholesterol, mmol/L—mean (SD)	3.1 (0.9)	3.0 (0.7)	0.54	3.0 (0.7)	2.8 (0.7)	0.28
HDL Cholesterol, mmol/L—mean (SD)	1.2 (0.3)	1.1 (0.3)	0.08	1.3 (0.3)	1.4 (0.5)	0.59
Triglycerides, mmol/L—median (IQR)	1.0 (0.7–1.3)	1.2 (0.9–1.7)	0.06	1.0 (0.7–1.3)	0.9 (0.6–1.4)	0.32
HS-CRP, mg/l—median (IQR)	3.6 (2.5–6.9)	3.1 (1.4–7.6)	0.25	2.7 (1.5–6.5)	1.2 (0.9–2.0)	< 0.01
*Follow-up characteristics*						
Age at follow-up, years—mean (SD)	36.3 (4.4)	36.5 (4.3)	0.83	40.6 (3.3)	38.0 (5.3)	0.08
Follow-up duration, months—median (IQR)	73.5 (61.3–80.4)	72.9 (65.4–80.4)	0.65	74.8 (71.0–82.2)	72.2 (69.8–82.2)	0.75
Pregnancy after randomization, no. (%)^b^	35 (70)	47 (77)	0.40	22 (100)	17 (100)	n.a.

a *P*-values of continuous outcomes based on student *t*-test or Mann–Whitney-U test. *P*-values of dichotomous and categorical outcomes are based on the Pearson Chi-Square test, the Fisher’s exact test or Fisher-Freeman-Halton exact test.

b Number of women that had a pregnancy of ≥24 weeks of gestation.

A comparison of baseline characteristics of participants and non-participants are presented in Supplementary table S1. Women who participated in the follow-up of the LIFEstyle study were more often Caucasian (94.6% vs. 85.7%; *P* = 0.01) and older at baseline (30.4 [4.1] vs. 29.6 [4.6] years; *P* = 0.07) than women who did not participate in the follow-up study.

### Outcomes

#### Primary analyses

In both studies, weight, BMI, waist and hip circumferences, blood pressure, fasting glucose, HOMA-IR, HbA1c, triglycerides, total cholesterol, LDL-C, HDL-C, hs-CRP, BFP and PWV were not statistically different between the intervention and control group (table 3).

**Table 3:** Cardiometabolic outcomes (change from baseline to 6-year follow-up and mean difference) in the intervention and control group of the LIFEstyle and RADIEL study

Cardiometabolic outcomes	LIFEstyle study						RADIEL study					
	*n*	Intervention	*n*	Control	MD^b^	BCa 99%	*n*	Intervention	*n*	Control	MD^b^^,^^c^	BCa 99%
	Δ^a^		Δ^a^		C.I.		Δ^a^		Δ^a^		C.I.	
Weight, kg—mean (SD)	50	−3.4 (14.2)	60	−1.5 (13.5)	−1.8	−8.8 to 4.7	22	2.9 (15.4)	17	−1.8 (9.3)	2.0	−10 to 11.8
BMI, kg/m^2^—mean (SD)	50	–0.5 (5.1)	60	0.0 (4.7)	–0.6	–2.7 to 1.6	22	1.1 (5.6)	17	–0.6 (3.2)	0.7	−3.5 to 4.2
Waist Circumference, cm—mean (SD)	48	–0.5 (12.5)	60	–0.3 (13.5)	–0.8	–6.1 to 4.6	21	7.1 (12.9)	17	8.1 (10.0)	–3.2	–14.3 to 8.0
Hip Circumference, cm—mean (SD)	49	–2.6 (9.9)	60	–2.5 (9.4)	–0.5	–5.0 to 3.6	21	2.9 (13.1)	17	–3.2 (6.6)	4.8	–4.6 to 13.8
Systolic Blood pressure, mmHg—mean (SD)	48	–3.9 (14.6)	60	–6.0 (15.3)	–0.2	–7.3 to 6.9	22	2.7 (11.1)	17	3.7 (15.0)	1.6	–11.2 to 17.5
Diastolic Blood pressure, mmHg—mean (SD)	48	1.6 (10.2)	60	0.5 (9.4)	–0.5	–5.1 to 4.4	22	–0.8 (8.4)	17	–2.8 (11.0)	4.1	–4.1 to 12.4
Fasting glucose, mmol/L—mean (SD)	36	0.0 (0.6)	52	0.0 (1.1)	–0.3	–0.7 to 0.2	22	–0.1 (0.7)	17	0.0 (0.5)	–0.2	–0.7 to 0.4
HOMA-IR—mean (SD)	35	–0.1 (2.3)	50	0 (2.6)	–0.4	–1.6 to 0.9	18	1.4 (2.8)	16	0.8 (2.2)	0.3	–2.3 to 2.6
Total Cholesterol, mmol/L—mean (SD)	36	–0.1 (1.0)	52	0.0 (0.8)	0.0	–0.5 to 0.5	22	–0.1 (0.7)	16	–0.1 (0.6)	–0.1	–0.6 to 0.5
LDL Cholesterol, mmol/L—mean (SD)	36	–0.2 (1.0)	52	–0.2 (0.7)	0.0	–0.4 to 0.4	22	0.0 (0.7)	16	0.2 (0.5)	–0.2	–0.8 to 0.4
HDL Cholesterol, mmol/L—mean (SD)	36	0.2 (0.3)	52	0.1 (0.3)	0.1	–0.0 to 0.3	22	0.1 (0.4)	16	0.0 (0.4)	0.1	–0.2 to 0.5
Triglycerides, mmol/L—mean (SD)	36	–0.3 (1.6)	52	–0.1 (0.6)	–0.3	–0.7 to 0.3	22	–0.1 (0.7)	16	0.1 (0.6)	–0.1	–0.6 to 0.4
HS-CRP, mg/l—mean (SD)	36	0.1 (4.5)	52	1.8 (5.6)	–1.6	–4.4 to 1.2	22	–0.5 (6.3)	17	–0.1 (2.5)	0.6	–3.5 to 5.9
HbA1c, mmol/mol—mean (SD)^d^	42	n.a.	52	n.a.	–1.7	–5.2 to 1.2	18	1.6 (4.7)	11	–0.3 (5.7)	1.2	–3.3 to 7.1
Fat percentage, %^e^	50	n.a.	60	n.a.	–0.5	–2.7 to 1.3	17	n.a.	16	n.a.	–1.1	–8.5 to 5.8
PWV, m/s^f^	37	n.a.	49	n.a.	0.1	–1.2 to 1.1	19	n.a.	16	n.a.	0.4	–0.8 to 1.7

a Change between baseline and follow-up.

b Mean differences between intervention and control group at follow-up based on linear regression models adjusted for baseline values, unless stated otherwise.

c Adjusted for age at baseline.

d No baseline value in LIFEstyle study, mean difference is unadjusted.

e No baseline value, mean difference is adjusted for BMI at baseline.

f No baseline value, mean difference is adjusted for pulse pressure at baseline.

Although the prevalence of MetS at follow-up was lower in the LIFEstyle intervention group compared with the control group (25.7 vs. 52.7%), adjustments for baseline prevalence showed no statistically significant difference between the intervention and control group (aOR: 1.11 95% CI 0.19–6.64). Also, no statistical significant difference in MetS prevalence at follow-up was found in the RADIEL study (aOR: 0.20 95% CI 0.01–2.8).

#### Subgroup analyses

Of the 50 women in the LIFEstyle intervention group, 22 women lost ≥5% body weight or reached a BMI <29 kg/m^2^ during the 6-month intervention period. These women had a lower BMI at baseline (34.2 ± 2.6 vs. 35.8 ± 3.2; *P* = 0.02), more often smoked (9 [40.9%] vs. 11 (18.0%); *P =* 0.03) and had been trying to conceive for a longer period of time (27 [IQR 19.5–40.25] vs. 16 [12.0–26.0] months; *P* = 0.04) compared with the control group. No other statistically significant differences were detected.

At follow-up, these successful women had lower weight (–8.1 kg; 99% BCa CI = −16.6 to −0.9), BMI (−3.3 kg/m^2^; 99% BCa CI = –6.5 to −0.8), smaller waist circumference (−8.2 cm; 99% BCa CI = −15.3 to −1.3), lower fasting glucose (−0.5 mmol/L; 99% BCa CI = −1.1 to −0.0), lower HbA1c (−4.1 mmol/mol; 99% BCa CI = −6.4 to −0.3), and higher HDL-C (0.3 mmol/L; 99% BCa CI =0.1–0.5) compared with controls (Supplementary table S2).

No subgroup analyses of successful intervention were performed for the RADIEL study, because only four of the RADIEL intervention women successfully reached the short-term weight goals.

#### Exploratory analyses

No statistically significant interaction effects were found for treatment group with pregnancy status after randomization or for treatment group with PCOS status on any of the continuous outcomes (all interaction *P* ≥0.05).

## Discussion

This is the first study reporting on the effects of preconception lifestyle interventions on long-term cardiometabolic health of overweight and obese women from two RCTs. Despite the positive short-term effects of the LIFEstyle preconception intervention, the six year follow-up of both the LIFEstyle and the RADIEL interventions did not show any effects on individual parameters of cardiometabolic health, nor on the prevalence of metabolic syndrome.^13^ However, in comparison to controls, women who successfully lost weight during the LIFEstyle intervention period had better long-term cardiometabolic outcomes in terms of smaller waist circumferences, lower weight, BMI, glucose and HbA1c, as well as higher HDL cholesterol concentrations.

The absence of an overall effect of the interventions on long-term cardiometabolic health is in line with the only other study with a similar follow-up duration after a lifestyle intervention in pregnancy.^29^ Other post-conception lifestyle interventions in overweight and obese women reported inconsistent effects on adverse maternal outcomes during and directly after pregnancy, and did not yet report on the long-term health of these women.^30–32^ Lifestyle is attained over time and is not easily changed without intrinsic motivation.^33^ The wish to have a child could be a strong motivator to improve lifestyle, but at the same time, the temporary nature of this motivator may explain the lack of long-term effects on cardiometabolic outcomes. After giving birth, mothers are exposed to the emotional post-partum period in which they adjust to their new role and often prioritize parenthood over their own wellbeing.^34^ However, we found no evidence that women who had an ongoing pregnancy (≥24 weeks) during the follow-up period had different long-term effects on cardiometabolic health compared with women who did not. The latter could be explained by the discouraging effect of persistent infertility on lifestyle improvement in this last group.^35^ In both scenario’s, an individualized relapse prevention phase following the actual intervention could help women to adhere to their improved lifestyle.^36^

The absence of long-term effects of the preconception lifestyle interventions on cardiometabolic health could also be explained by the high-risk profile of the study populations, who might need more intensive and prolonged lifestyle interventions for sustainable effects on cardiometabolic health. In the RADIEL study, 67% of the women in the current follow-up study were diagnosed with GDM in their index pregnancy, leading to additional lifestyle advice and intensive follow-up from the healthcare system in both treatment groups. This regular care might have overshadowed the effect of the preconception lifestyle intervention, diminishing the potential differences between the intervention and control group. In the LIFEstyle study, 39% of the women were diagnosed with PCOS. Intrinsic insulin resistance, alteration in appetite regulation and abdominal fat distribution can challenge weight management in women with PCOS.^37^^,^^38^ However, the relatively high prevalence of women with PCOS in the LIFEstyle study could not explain the absence of long-term effects since no interaction effect was observed between treatment group and PCOS status on cardiometabolic outcomes.

Timing and duration of lifestyle interventions are possible determinants of successful lifestyle change, but in this study we found no such evidence.^39^ Scheduling the intervention solely before (LIFEstyle) compared with before, during, and after pregnancy (RADIEL) both failed to provide beneficial cardiometabolic effects in the long run.

Both studies had considerable attrition (LIFEstyle study: 80.7%; RADIEL study: 67.8%), leading to limited statistical power to detect relevant differences and introduction of potential selection bias. To diminish potential confounding effects of selection on the outcome assessments, the regression analyses were adjusted for baseline values. Women of the LIFEstyle study follow-up were more likely to participate if they were Caucasian and slightly (0.8 year) older at randomization. Although cardiometabolic plasticity decreases with age, it is unlikely that this small difference in age explains our null-findings.^40^ Similar selection was not found for the RADIEL study (Supplementary table S1). Because of the high percentage of Caucasian women in both studies, it should be noted that our findings should not be generalized to women of other ethnicities.

Short-term success is usually a good indicator for long-term effects.^14^^,^^35^ Although we did not find overall effects, women who successfully lost weight during the LIFEstyle intervention, did have better cardiometabolic health 6 years after the intervention compared with control women. Future studies should therefore investigate determinants of a successful lifestyle intervention, in order to identify women who would benefit the most and to make tailored approaches more effective.

## Supplementary Material

Supplementary Tables
