# Supplementary material for: Long-term effects of a preconception lifestyle intervention on cardiometabolic health of overweight and obese women
Source: Eur J Public Health. 2018 Oct 30;29(2):308–14. doi: 10.1093/eurpub/cky222 (PMC6427693; doi:10.1093/eurpub/cky222)
Supplement: Supplementary Tables [file cky222_supplementary_tables.doc]

Supplementary Table 1. Comparison of baseline characteristics between non-participants and participants of the LIFEstyle and RADIEL study.

| **Baseline characteristics** | | | | | | |
| --- | --- | --- | --- | --- | --- | --- |
| **Variable** | **LIFEstyle study** | | | **RADIEL study** | | |
|  | **Non participants N=463** | **Participants N=111** | **P-value ^a^** | **Non participants N=82** | **Participants N=39** | **P-value ^a^** |
| **Age, years – mean (SD)** | 29.6 (4.6) | 30.4 (4.1) | 0.07 | 32.2 (4.6) | 33.1 (4.4) | 0.30 |
| **Weight, kg – mean (SD)** | 103.3 (13.3) | 103.2 (12.1) | 0.96 | 95.2 (13.5) | 96.3 (15.5) | 0.69 |
| **BMI, kg/m^2^ – mean (SD)** | 36.1 (3.5) | 35.7 (3.0) | 0.19 | 34.5 (3.8) | 34.7 (4.5) | 0.79 |
| **Ethnicity – no. Caucasian (%)** | 397 (85.7) | 105 (94.6) | 0.01 | 86 (100) | 39 (100) | n.a. |
| **Education – no. (%)** |  |  | 0.23 |  |  | 0.34 |
| **Basic education** | 26 (5.9) | 1 (0.9) |  | 5 (6.1) | 0 (0) |  |
| **Vocational education** | 96 (21.7) | 23 (21.7) |  | 22 (26.8) | 6 (15.8) |  |
| **Secondary education** | 10 (2.3) | 2 (1.9) |  | 8 (9.8) | 5 (13.2) |  |
| **Vocational & Secondary education** | 214 (48.3) | 52 (49.1) |  | 29 (35.4) | 16 (42.1) |  |
| **Higher education** | 97 (21.9) | 28 (26.4) |  | 18 (22.0) | 11 (28.9) |  |
| **Alcohol use – no. (%)** | 139 (37.0) | 38 (37.6) | 0.90 | 44 (55.7) | 23 (60.5) | 0.62 |
| **Current smoker – no. (%)** | 111 (24.2) | 25 (22.7) | 0.75 | 10 (12.2%) | 2 (5.1) | 0.33 |
| **Nulliparous – no. (%)^b^** | 355 (76.8) | 86 (77.5) | 0.89 | 20 (24.4) | 7 (17.9) | 0.43 |

^a^ P-values of continuous outcomes based on student t-test or Mann-Whitney-U test. P-values of dichotomous and categorical outcomes are based on the Pearson Chi-Square test, the Fisher’s exact test or Fisher-Freeman-Halton exact test.

Supplementary Table 2. Cardiometabolic outcomes (change from baseline to six year follow-up and mean difference) of 22 participants that lost ≥ 5% body weight or lowered BMI <29 during the LIFEstyle intervention and the control group.

|  | **LIFEstyle study** | | | | **Model 1** | | **Model 2** | |
| --- | --- | --- | --- | --- | --- | --- | --- | --- |
| **Cardiometabolic outcome** | **N** | **Successful Intervention  Δ ^a^** | **N** | **Control Δ ^a^** | **MD ^b^** | **BCa 99% C.I.** | **MD ^f^** | **BCa 99% C.I.** |
| **Weight, kg – mean (SD)** | 22 | -7.6 (13.1) | 60 | -1.5 (13.5) | -7.2 | -15.9 – 0.2 | -8.1 ^g^ | -16.6 – -0.9 |
| **BMI, kg/m^2^ – mean (SD)** | 22 | -2.1 (4.7) | 60 | 0.0 (4.7) | -3.0 | -5.9 – -0.3 | -3.3 | -6.5 – -0.8 |
| **Waist Circumference, cm – mean (SD)** | 22 | -4.2 (11.5) | 60 | -0.3 (13.5) | -7.1 | -13.4 – -0.8 | -8.2 ^g^ | -15.3 – -1.3 |
| **Hip Circumference, cm – mean (SD)** | 21 | -4.6 (10.2) | 60 | -2.5 (9.4) | -4.1 | -9.6 – 1.7 | -4.3 ^g^ | -10.7 – 2.5 |
| **Systolic Blood pressure, mmHg – mean (SD)** | 22 | -7.1 (12.9) | 60 | -6.0 (15.3) | -2.4 | -11.8 – 6.3 | -3.5 | -12.7 – 6.3 |
| **Diastolic Blood pressure, mmHg – mean (SD)** | 22 | -0.0 (10.0) | 60 | 0.5 (9.4) | -2.0 | -7.6 – 4.8 | -2.5 | -8.5 – 3.1 |
| **Fasting glucose, mmol/L – mean (SD)** | 16 | -0.3 (0.4) | 52 | 0.0 (1.1) | -0.5 | -0.9 – -0.0 | -0.5 | -1.1 – -0.0 |
| **HOMA-IR – mean (SD)** | 16 | -0.5 (1.7) | 50 | 0.0 (2.6) | -0.9 | -2.2 – 0.8 | -0.8 | -2.1 – 0.7 |
| **Total Cholesterol, mmol/L – mean (SD)** | 16 | 0.0 (1.0) | 52 | 0.0 (0.8) | 0.1 | -0.7 – 0.8 | 0.1 | -0.7 – 0.7 |
| **LDL Cholesterol, mmol/L – mean (SD)** | 16 | 0.0 (1.1) | 52 | -0.2 (0.7) | 0.1 | -0.5 – 0.8 | 0.0 | -0.6 – 0.7 |
| **HDL Cholesterol, mmol/L – mean (SD)** | 16 | 0.3 (0.3) | 52 | 0.1 (0.3) | 0.2 | 0.0 – 0.4 | 0.3 | 0.1 – 0.5 |
| **Total Triglycerides, mmol/L – mean (SD)** | 16 | -0.7 (2.3) | 52 | -0.1 (0.6) | -0.5 | -0.9 – 0.2 | -0.5 | -1.0 – 0.1 |
| **HS-CRP, mg/l – mean (SD)** | 16 | 1.0 (3.9) | 52 | 1.8 (5.6) | -0.8 | -4.0 – 2.4 | -0.5 | -5.0 – 3.2 |
| **HbA1c, mmol/mol** ^c^ | 19 | n.a. | 52 | n.a. | -3.0 ^c^ | -6.4 – -0.3 | -4.1 | -9.1 – -0.8 |
| **Fat percentage, %** ^d^ | 22 | n.a. | 60 | n.a. | -2.0 ^d^ | -4.6 – 0.5 | -1.6 | -4.5 – 0.9 |
| **Pulse wave velocity, m/s** ^e^ | 18 | n.a. | 49 | n.a. | 0.3 ^e^ | -1.2 – 1.7 | 0.6 | -1.0 – 2.1 |

^a^ Change between baseline and follow-up. ^b^ Mean differences between intervention and control group based on linear regression models adjusted for baseline values (model 1), unless stated otherwise. ^c^ No baseline HbA1c, unadjusted. ^d^ No baseline fat percentage, adjusted for BMI at baseline. ^e^ No baseline pulse wave velocity, adjusted for pulse pressure at baseline. ^f^ Mean differences between intervention and control group based on linear regression models. In model 2, BMI, smoking status and duration of infertility at baseline are added as covariates to model 1, unless stated otherwise. ^g^ BMI at baseline was not added as covariate to model 2.
